# Voiding symptoms aggravate with decreasing stromal/epithelial ratio and increasing glandular-epithelial content in patients undergoing laser enucleation for benign prostatic hyperplasia, independently from prostate size

**DOI:** 10.1371/journal.pone.0345306

**Published:** 2026-03-24

**Authors:** Patrick Keller, Sheng Hu, Wenbin Zhu, Yajie Xu, Laurenz Berger, Philip Nicola, Philipp Weinhold, Alexander Tamalunas, Christian G. Stief, Martin Hennenberg

**Affiliations:** Department of Urology, LMU University Hospital, LMU Munich, Munich, Germany; Jan Biziel University Hospital No 2 in Bydgoszcz: Szpital Uniwersytecki Nr 2 im dr Jana Biziela w Bydgoszczy, POLAND

## Abstract

**Introduction:**

Benign prostatic hyperplasia (BPH) includes epithelial, stromal and mixed hyperplasia, but their specific contributions to voiding symptoms and prostate volume (PV) are unknown. Here, we examined relationships of symptoms and PV with stromal and epithelial markers in patients undergoing laser enucleation for BPH.

**Methods:**

Tissues were obtained from holmium or thulium laser enucleation of the prostate (n = 146 patients). Expressions of the smooth muscle marker calponin-1 (CNN1) and the glandular-epithelial cell marker keratin-19 (KRT19) were assessed by RT-PCR and Western blot, and analyzed for correlation with international prostate symptom score (IPSS), maximum urinary flow rate (Q_max_), and PV.

**Results:**

The ratio of CNN1/KRT19 mRNA correlated positively with Q_max_ (r = 0.3809, p = 0.0263), and by trend negatively with IPSS (r = −0.2161, p = 0.0944). Accordingly, the IPSS increased with keratin protein expression (r = 0.4244, p = 0.0307), while the Q_max_ tended to correlate negatively with keratin expression (r = −0.2058, p = 0.4999). PV correlated negatively with CNN1 mRNA expression (r = −0.205, p = 0.0405). The inverse correlation of CNN1 with PV persisted in patients without catheterization (r = −0.2568, p = 0.0457), but was lacking in catheterized patients after separated analyses.

**Conclusions:**

Voiding symptoms in patients undergoing laser enucleation for BPH aggravate with increasing keratin content. Symptoms in patients needing surgery for BPH depend rather on glandular-epithelial hyperplasia, but not on stromal hyperplasia, what might explain why these patients are refractory to treatment with α_1_-blockers.

## Introduction

Voiding symptoms in benign prostatic hyperplasia (BPH) may be caused by an urethral obstruction, imparted by an elevated prostate smooth muscle tone, by hyperplastic growth, or both [1]. Medical treatment is based on antagonists for α_1_-adrenoceptors and the phosphodiesterase-5 inhibitor tadalafil, both reducing symptoms by prostate smooth muscle relaxation, and on 5α-reductase inhibitors to prevent progression and complications by inhibition of prostate growth [2]. Surgery is required if complications are experienced or imminent, including recurrent or refractory urinary retention, overflow incontinence, recurrent urinary tract infections, bladder stones or diverticula, treatment-resistant macroscopic hematuria due to BPH, or dilatation of the upper urinary tract due to urethral obstruction, with or without renal insufficiency, as defined in the current guidelines of the European Association of Urology [2]. In addition, surgical intervention for BPH is considered when symptoms or postvoid residual urine persist despite medical therapy, or when patients decline pharmacological treatment but still seek active management [2,3]. Typically, candidates for BPH surgery present with severe symptoms, whereas α_1_-adrenoceptor antagonists are recommended for patients with moderate to severe symptoms [2]. Although α_1_-blockers are the first line option, their effectiveness is limited and the rate of non-responders mounts to one-third [4]. Decreases in international prostate symptom scores (IPSS) by α_1_-blockers may not exceed 25% in 30–35% of patients, so that up to 69% of patients are disappointed, contributing to discontinuation rates around 65% within 12 months [5]. Discontinuation results in progression, complications, hospitalization and surgery for BPH [6,7]. For decades, transurethral resection of the prostate (TURP) was considered the surgical standard treatment for BPH, while holmium and thulium laser enucleation (HoLEP and ThuLEP) are emerging alternatives [2,3,8,9].

Tissue composition in BPH is highly heterogenous, and may include glandular, stromal, and mixed hyperplasia [10]. However, their single contributions to clinical conditions including voiding symptoms, prostate size, responsiveness to medical treatment or the need for surgery are poorly understood [10]. It has been supposed, that lacking knowledge on phenotypic heterogeneity of BPH impaired clinical developments [10]. Drug efficacy in clinical trials has been addressed for overall study populations and subgroups, but never separately for morphological phenotypes [10]. Major cell types in the prostate are glandular epithelial cells, and smooth muscle cells constituting the stroma. Prostatic α_1_-adrenoceptors are expressed on stromal smooth muscle cells [4]. Consequently, clinical responses to α_1_-blockers are proportional to the percentage of smooth muscle cells and may be adequate in stromal hyperplasia, but insufficient in patients with predominant glandular hyperplasia [10]. Though the glandular epithelium is again variable and may contain three different types of epithelial cells, all of them express cytokeratins, including keratin-19 [10,11]. Smooth muscle cells in turn may exhibit different phenotypes, but consistently express calponin-1 [12].

Morphometric studies assessed the prevalence of histological phenotypes in BPH, using tissues from TURP, from biopsies for suspected prostate cancer, or from radical prostatectomy for prostate cancer. However, relationships of histological findings and tissue composition by stromal and epithelial compartments, to voiding symptoms and urodynamics have not been reported [10]. Moreover, the conclusiveness of findings was considered limited, owing to small sample sizes of biopsies, to intraprostatic heterogeneities, and tissue traumatization [10]. Here, we examined expression of calponin-1 and keratin-19 in tissues from laser enucleation for BPH, and their correlations with IPSS, maximum urinary flow rate (Q_max_) and prostate volume (PV).

## Materials and methods

### Tissues from HoLEP and ThuLEP

Morcellated prostate tissues were collected from January 9^th^, 2023, to August 2^nd^, 2024 from surgery for BPH by HoLEP and ThuLEP performed at our department. This study was carried out in accordance with the Declaration of Helsinki of the World Medical Association. All procedures were performed in compliance with relevant laws and institutional guidelines and have been approved by the ethics committee of the Ludwig-Maximilians University, Munich, Germany (approval number 22−0608). Written informed consent was obtained from all patients, and the privacy rights of human subjects have been observed. Samples and patients’ data were collected and analyzed pseudonymized, and were anonymized after merge of data from tissue analyses with clinical data. HoLEP was performed in a three-lobe technique using the VersaPulse^®^ 100W Holmium Laser (Lumenis Ltd., Yokneam, Israel), as recently described [13]. For transport and interim storage, tissues were placed in Custodiol^®^ solution (Köhler, Bensheim, Germany) immediately after extraction. Within 2 h following extraction, about 5 pieces per macerate (i.e., per prostate or patients) were selected from the sampled tissue for RT-PCR, and about 10 pieces for Western blot analyses, which were pooled to single samples, shock frozen in liquid nitrogen, and stored at −20°C (not longer than 4 weeks) until analyses were initiated.

### Clinical data

Clinical data for prostate volume (ml), IPSS, Q_max_ (ml/sec) assessed by uroflowmetry and for catheterization were obtained during preoperative, routine diagnoses for voiding symptoms and the need for surgery. Tissues from 100 patients were analyzed by RT-PCR, and from 46 patients by Western blot. In the cohort analyzed for mRNA expression by RT-PCR, data on prostate volume was available from all patients (n = 100), and data on IPSS from 61 patients (57 without catheterization, 4 catheterized). Before surgery, 38 of these patients were catheterized due to urinary retention, while 61 were not and information for catheterization was unavailable from one patient. Q_max_ was available from 34 patients (all without catheter) in the RT-PCR cohort. In the cohort analyzed for protein expression by Western blot, data on prostate volume was available from 45 patients, and data on IPSS from 26 patients (24 without catheterization, 1 catheterized, 1 without information on catheterization). Q_max_ was available from 13 patients (all without catheter) in the Western blot cohort.

### RT-PCR

RNA was isolated from frozen tissues using the RNeasy Mini Kit (Qiagen, Hilden, Germany) based on the manufacturer’s instructions. For isolation, 30 mg of frozen tissues was homogenized using the FastPrep^®^-24 system with matrix A (MP Biomedicals, Illkirch, France). RNA concentrations were measured spectrophotometrically. cDNA was synthesized using 500 ng of isolated RNA using a Reverse Transcription System (Promega, Madison, WI, United States). Real-time PCR (RT-PCR) was performed with a Roche Light Cycler (Roche, Basel, Switzerland). Primers (with RefSeq accession no) for calponin-1 (CNN1, NM_001299), cytokeratin-19 (KRT19, NM_002276) and glyceraldehyde-3-phosphate dehydrogenase for housekeeping (GAPDH, NM_002046) were purchased from Qiagen (Hilden, Germany). PCR reaction volume was 10 μl, which included 5 µl FastStart DNA MasterPlus SYBR Green I (Roche, Basel, Switzerland), 1 μl primer, 1.5 μl PCR grade water and 2.5 μl sample. Denaturation was performed at 95°C for 10 min, and amplification with 40 cycles, each including 10 s at 95°C, 10 s at 60°C, 15 s at 72°C. PCR product quality was demonstrated by post-PCR melt curve analysis. All samples were determined in duplicate and presented as means of these two replicates. Results were expressed using the 2^−ΔCt^ method, where number of cycles (Ct) at which the fluorescence signal exceeded a defined threshold for GAPDH was subtracted from Ct values for targets (Ct_target_-Ct_GAPDH_ = ΔCt), and values were calculated as 2^−ΔCt^ and normalized to the mean values of corresponding controls.

### Western blot

Frozen tissues were homogenized in a buffer containing 25 mM Tris/HCl, 10 μM phenylmethanesulfonyl fluoride, 1 mM benzamidine, and 10 μg/ml leupeptine hemisulfate, using the FastPrep^®^-24 system with matrix A. After centrifugation (20,000 g, 4 min), supernatants were assayed for protein concentration using the Dc-Assay kit (Biorad, Munich, Germany) and boiled for 10 min with sodium dodecyl sulfate (SDS) sample buffer (Roth, Karlsruhe, Germany). Samples (20 μg/lane) were subjected to SDS-polyacrylamide gel electrophoresis, and proteins were blotted on Protran^®^ nitrocellulose membranes (Schleicher & Schuell, Dassel, Germany). For blockage of unspecific binding sites, membranes were blocked with phosphate-buffered saline (PBS) containing 5% milk powder (Roth, Karlsruhe, Germany) overnight. Subsequently, membranes were washed three times (each time for 5 min) with PBS containing 0.1% Tween 20 (PBS-T), followed by incubation with mouse monoclonal anti pan-cytokeratin (sc-8018), mouse monoclonal anti calponin 1/2/3 (sc-136987), or mouse monoclonal anti-β-actin antibody (sc-47778) (Santa Cruz Biotechnology, Santa Cruz, CA, USA) for 90 min. Primary antibodies were diluted 1:500 in PBS-T containing 5% milk powder. Subsequently, membranes were washed with PBS-T (4 times, each time for 5 min). For detection of calponin and pan-cytokeratin, membranes were then incubated with secondary biotinylated horse anti-mouse IgG (BA-2000) (Vector Laboratories, Burlingame, CA, USA) diluted 1:1500 in PBS-T containing 5% milk powder, washed again with PBS-T (4 times, each time for 5 min), incubated with avidin and biotinylated horseradish peroxidase (HRP) from the “Vectastain ABC kit” (Vector Laboratories, Burlingame, CA, USA) both diluted 1:800 in PBS and washed again with PBS-T (4 times, each time for 5 min). For detection of β-actin, membranes were incubated with secondary goat anti mouse IgG, H&L Chain Specific Peroxidase Conjugate (401,215) (Sigma-Aldrich, Munich, Germany) diluted 1:3000 in PBS-T containing 5% milk powder, and washed again with PBS-T (4 times, each time for 5 min). Blots were developed with SuperSignal west pico plus chemiluminescent substrate (34,577) (ThermoScientific, Rockford, United States) and imaged using the ChemiDoc XRS+ System (Bio-Rad, Hercules, CA, United States), with constant exposure times of 60 seconds for calponin and pan-cytokeratin, and 10−20 seconds for β-actin. Intensities of resulting bands for β-actin (detected bands matching the molecular weight of 41.7 kDa), calponin (detected bands matching the molecular weight of 33 kDa) and pan-cytokeratin (all detected bands between 37−50 kDa) were quantified densitometrically using ImageJ (National Institutes of Health, Bethesda, Maryland, United States), and bands for calponin and cytokeratins were normalized to β-actin in corresponding samples. The range of 37−50 kDa in pan-cytokeratin detection covers most, if not all acidic keratins in soft keratinization, including keratin-18 and −19 [14], which are major keratins in the glandular epithelium.

### Correlation analyses and statistical analyses

Pearson correlation analyses, calculations of means and 95% confidence intervals (95% CIs), normality tests and statistical tests were performed using Graphpad Prism (version 6, GraphPad Software Inc., San Diego, CA, United States). P values <0.05 were considered statistically significant. Correlation coefficients (r) and p values in figures are indicated if p values <0.1 were observed, while all r and p values are reported in the text. Data for 2^-ΔCt^ values are summarized in scatter plots containing all single values together with means, and are reported with 95% CI in the text. For comparison of 2^-ΔCt^ values between patients with and without catheterization, distribution within groups was examined by D’Agostino & Pearson omnibus normality test, and groups were compared by unpaired, two-tailed t-test if normally distributed in both groups, or by Mann Whitney test if values were not normally distributed in at least one of both groups. No data were excluded from each analysis. Despite definite aims and a study plan, the present study and analyses have exploratory character and were not designed to test a pre-specified or statistical null hypothesis. Features of a typical hypothesis-testing study are lacking, including the definition of a tested hypothesis, or a study plan based on the biometric calculation of group sizes [15]. Owing to the exploratory study design, p values reported here are descriptive, but not hypothesis-testing [15].

## Results

### Patients

**Fig 1:**
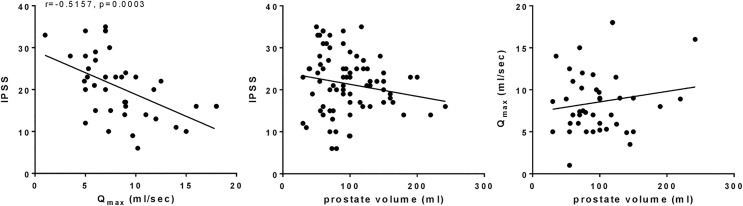
Correlation of IPSS with Q_max_, of IPSS with prostate volume, and of Q_max_ with prostate volume in the examined patient population (n = 146 patients). Laser-enucleated samples from 100 patients were subsequently analyzed by RT-PCR, and 46 samples by Western blot analyses. Data for prostate volume was available from 145 patients, data for IPSS from 87 patients, and for Q_max_ from 47 patients. Correlation analyses were performed by Pearson correlation analyses.

Across the whole study population (n = 146 patients), IPSS correlated negatively with Q_max_ (r = −0.5157, p = 0.0003) (fig. 1). Neither IPSS nor the Q_max_ correlated with prostate size (r = 0.1575, p = 0.2903 for Q_max_ vs. prostate volume; r = −0.1717, p = 0.1118 for IPSS vs. prostate volume) (Fig 1).

### Detection of calponin and cytokeratin mRNA and protein expression

**Fig 2:**
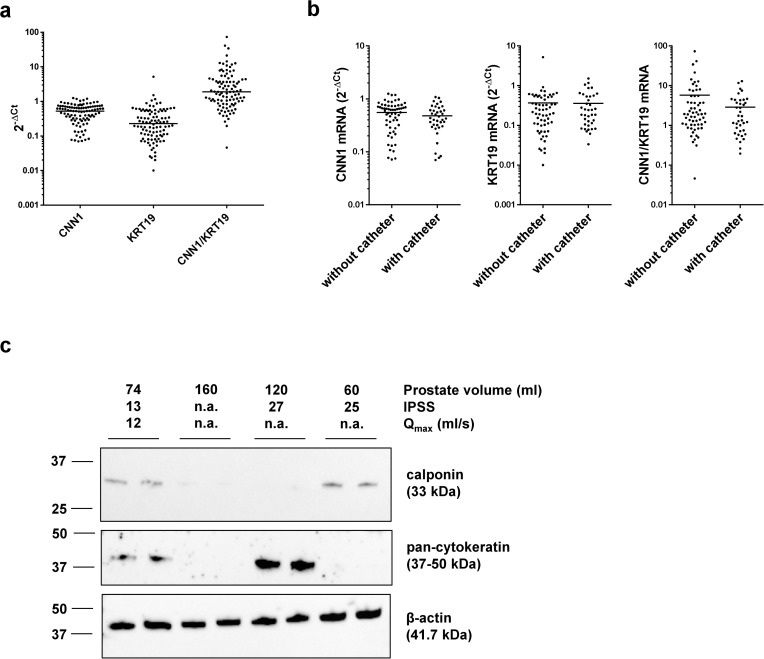
Detection of calponin and cytokeratin expression in laser-enucleated prostate tissues. Laser-enucleated prostate tissues were subjected to detection of calponin-1 (CNN1) and keratin-19 (KRT19) mRNA expression by RT-PCR **(a, b)**, and of calponin and pan-cytokeratin protein expression by Western blot analyses **(c)**. Shown are all single values from RT-PCR in **(a)**, which were compared between catheterized (n = 38) and non-catheterized patients (n = 61) in **(b)** (CNN1 compared by unpaired, two-tailed t-test; KRT19 and CNN1/KRT19 compared by Mann Whitney test; all insignificant). Representative Western blots are shown in **(c)**. All three blots are from the same sample set. Numbers on the left indicate positions of molecular weight markers. Data for prostate volume, IPSS and Q_max_ from the corresponding patients are indicated on the top (n.a., information not available).

Tissues from 100 patients were analyzed by RT-PCR, and from 46 patients by Western blot. CNN1 and KRT19 mRNA were both detectable in tissue from each patient (Fig 2a). KRT19 mRNA expression did not differ between patients without and with catheterization (2^-ΔCt^ 0.01 [0.19–0.54] without catheter, 0.03 [0.24–0.47] with catheter) (Fig 2b). CNN1 mRNA expression was again similar between patients without and with catheterization (2^-ΔCt^ 0.55 [0.48–0.63] without catheterization, 0.48 [0.39–0.56] with catheter) (Fig 2b). The CNN1/KRT19 ratio was slightly (though, not significantly) lower in catheterized compared to non-catheterized patients (5.7 [2.8–8.7] without catheter, 2.9 [1.9–3.9] with catheter) (Fig 2b).

Western blot analyses revealed bands with sizes matching the expected molecular weights of calponin (33 kDa), and bands between 37−50 kDa matching the molecular weights of keratin-19 (44 kDa) (Fig 2c). Band intensities varied between patients (Fig 2c). Partly, different phenotypes became visible even without quantification, including samples consisting predominantly of stroma (Fig 2c, right sample, reflected by detectable calponin, but undetectable keratin), consisting predominantly of glandular follicles (Fig 2c, second sample from right, reflected by strong keratin signal, but undetectable calponin), with weak levels of calponin and keratins (Fig 2c, second sample from left), or mixed composition (Fig 2c, left sample).

### Correlation of CNN1 and KRT19 mRNA with prostate volume, IPSS and Q_max_

**Fig 3:**
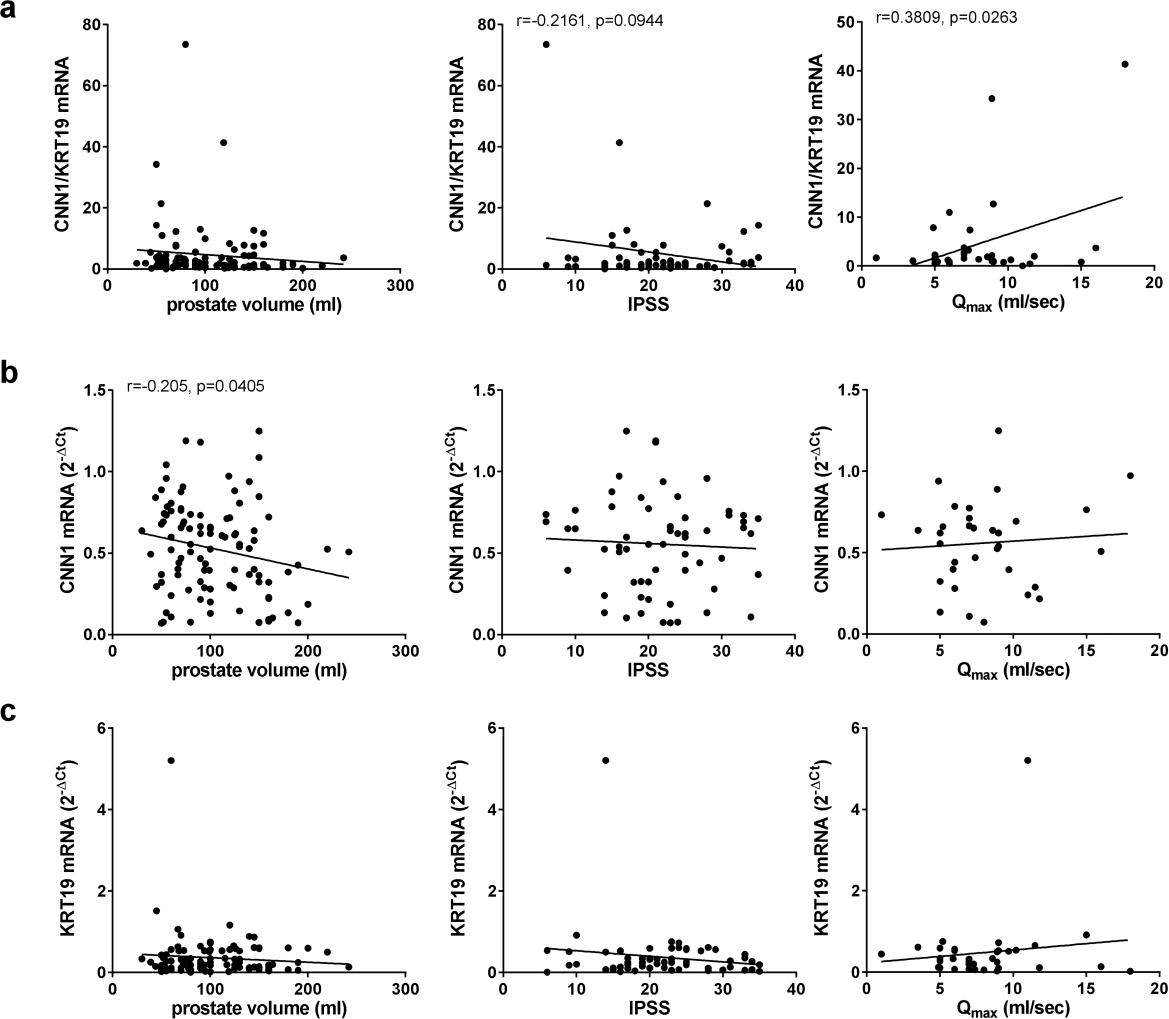
Expression of calponin-1 and keratin-19 mRNA in laser-enucleated prostate tissues, and correlation with IPSS, Q_max_ and prostate volume. Laser-enucleated tissues (n = 100 patients) were subjected to RT-PCR for calponin-1 (CNN1) and keratin-19 (KRT19). The ratio of 2^-ΔCt^ values for CNN1 and KRT19 (CNN1/KRT19) **(a)**, 2^-ΔCt^ values for CNN1 **(b)**, and 2^-ΔCt^ values for KRT19 **(c)** were subjected to Pearson correlation analyses with prostate volume, IPSS and Q_max_ in these patients.

The ratio of CNN1/KRT19 mRNA correlated positively with Q_max_ (r = 0.3809, p = 0.0263), and by trend inversely with the IPSS (r = −0.2161, p = 0.0944), but not with prostate volume (r = −0.1079, p = 0.2851) (Fig 3a). CNN1 mRNA alone did not correlate with Q_max_ (r = 0.0787, p = 0.658) or IPSS (r = −0.0548, p = 0.6751), but negatively with prostate volume (r = −0.2052, p = 0.0405) (Fig 3b). KRT19 mRNA alone did not correlate with Q_max_ (r = 0.1268, p = 0.4748), IPSS (r = −0.1489, p = 0.2522) or prostate volume (r = −0.08976, p = 0.3745) (Fig 3c).

**Fig 4:**
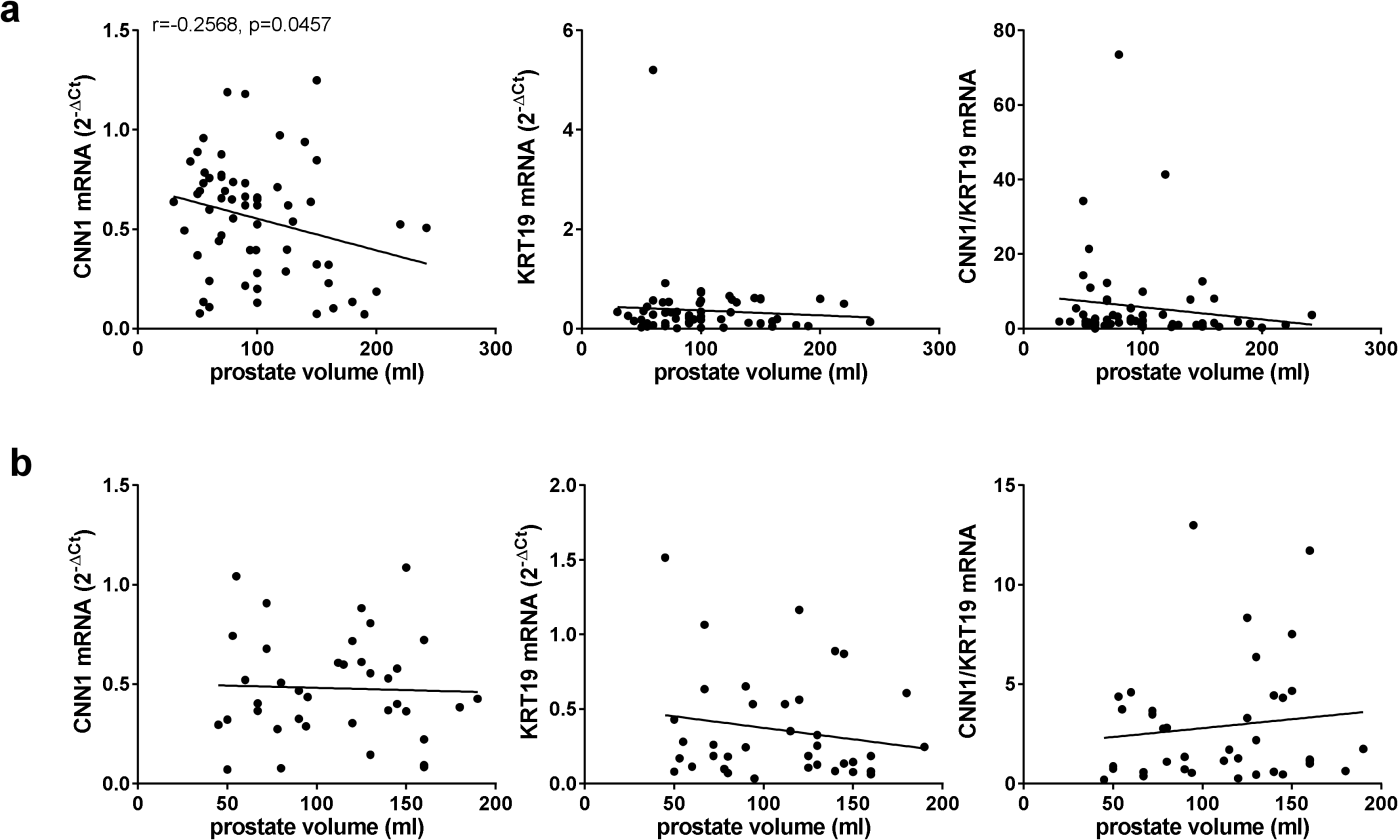
Correlation of CNN1 and KRT19 mRNA expression with prostate volume in patients with and without catheterization. Laser-enucleated tissues (n = 100) were subjected to RT-PCR for calponin-1 (CNN1) and keratin-19 (KRT19). Separate Pearson correlation analyses for mRNA expression with prostate volume were performed for patients without catheterization **(a)** and with catheterization for urinary retention **(b)**.

The inverse correlation of CNN1 with prostate volume persisted in patients without catheterization (r = −0.2568, p = 0.0457) (Fig 4a), but was lacking in catheterized patients (r = −0.03514, p = 0.8295), after separated analyses for subgroups (Fig 4b). KRT19 did not correlate with prostate volume in any of both subgroups (r = −0.06966, p = 0.5937 without catheter; r = −0.1787, p = 0.2832 with catheter) (Fig 4). The CNN1/KRT19 ratio showed no significant correlations, but opposing trends after separated analyses for subgroups (r = −0.1351, p = 0.2993 without catheter; r = 0.1192, p = 0.476 with catheter) (Fig 4).

### Correlation of calponin and cytokeratin protein expression with prostate volume, IPSS and Q_max_

**Fig 5:**
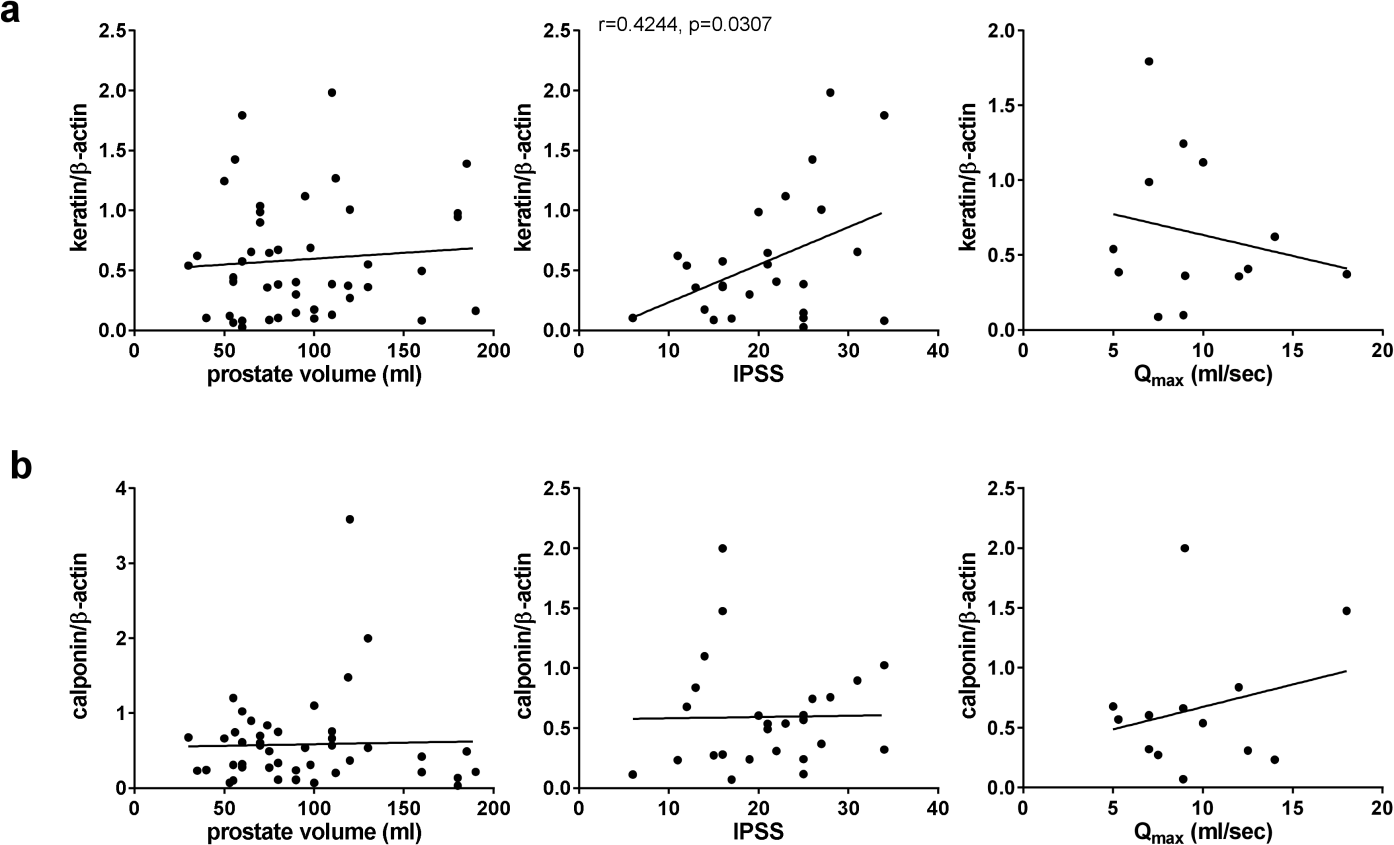
Protein expression of calponin and cytokeratins in laser-enucleated prostate tissues, and correlation with IPSS, Q_max_ and prostate volume. Laser-enucleated tissues (n = 46 patients) were subjected to Western blot analyses for cytokeratins, calponin and β-actin. Intensities of bands from 37-50 kDa in pan-cytokeratin expression **(a)**, and matching the expected molecular weight of calponin (33 kDa) **(b)** were referred to band intensities of β-actin in the same samples, and subjected to Pearson correlation analyses with prostate volume, IPSS and Q_max_ in these patients.

The IPSS increased with keratin expression (r = 0.4244, p = 0.0307) (Fig 5a). The Q_max_ decreased by trend with keratin expression (r = −0.2058, p = 0.4999) (Fig 5a). Prostate volume was neither related to keratin expression (r = 0.0833, p = 0.58659) (Fig 5a), nor to calponin expression (r = 0.0283, p = 0.8535) (Fig 5b). The IPSS did not correlate with calponin expression (r = 0.0166, p = 0.9357) (Fig 5b). The Q_max_ increased insignificantly with calponin expression (r = 0.2597, p = 0.3949) (Fig 5b).

## Discussion

Our findings from RT-PCR and Western blot consistently suggest that epithelial, rather than stromal hyperplasia may account most for voiding symptoms in patients undergoing laser enucleation for BPH. Thus, the Q_max_ increased with the calponin-1/keratin-19 mRNA ratio, while the IPSS showed a trend for an inverse correlation, and increased with the cytokeratin protein expression. Most, if not all patients undergoing surgery for BPH are refractory for medical treatment with BPH-specific drugs [2,3]. Alpha1-blockers act on smooth muscle cells in the stroma and are the first-line option for medical improvement of voiding symptoms [2,4]. In view of our finding that symptoms in patients needing surgery for BPH may be attributed to glandular instead of stromal hyperplasia, it appears plausible, that these patients do not respond sufficiently to α_1_-blockers. Although tissue composition by stromal and glandular compartments in BPH has been studied, it has to the best of our knowledge rarely or possibly never before been related to symptoms or to urodynamic characteristics.

BPH encompasses different phenotypes, including stromal, glandular-epithelial and mixed hyperplasia [10]. Their specific prevalence in BPH has been studied, but their contributions to symptoms and complications, or to the necessity of surgery remain unknown [10]. It has been supposed, that phenotypes may decide about patients’ responsiveness to BPH-specific drugs [10]. The efficacy of α_1_-blockers to reduce voiding symptoms obviously depends on the percentage of stromal compartments and smooth muscle content [16]. In the past, phenotypic heterogeneity seriously affected developments and research addressing human BPH [10]. In view of the limited efficacy of BPH drugs across whole populations, together with adherent economic considerations and the poor knowledge on tissue compositions in BPH, research efforts addressing phenotype heterogeneity in BPH have been demanded, to improve diagnosis and to allow individual and cost-optimized treatment [10].

Pioneer views assuming that BPH predominantly depends on stromal hyperplasia have been challenged. Stromal hyperplasia may be in fact, though not necessarily predominant in early stages of BPH, or in subgroups without severe symptoms and without need for surgery. An early morphometric study reported percentages of 62%, 15% and 23% for stroma, epithelium and glandular lumen in tissues from 33 patients receiving surgery or drug treatment for symptomatic BPH, and of 54%, 21% and 25% in tissues from 6 patients with asymptomatic BPH undergoing surgery for prostate cancer [17]. Another morphometric analysis, of biopsies before initiation of drug treatment for symptomatic BPH in 41 patients found a composition of 84% stroma, 9% epithelium and 2.6% glandular lumen [18]. Similarly, a recent study still observed stromal-dominated hyperplasia (i. e., the stroma covered ≥50% of microscopic fields) in 70.1% of 67 patients undergoing TURP, and epithelial-dominated hyperplasia (stroma covered <50% of areas) in 29.9% of these patients [19]. Quantitative image analysis of tissues from patients undergoing radical prostatectomy for prostate cancer (IPSS 13.4) revealed a balanced ratio, of 50.4% stromal and 49.6% glandular (19.9% epithelium plus 29.7% lumina) compartments in “inner” zones of the prostates, and a 25% higher epithelial content in the “outer” prostate [20]. These findings suggesting predominance of stromal hyperplasia are not necessarily conflicting with our results, as they simply described tissue composition, but no correlations with symptoms as in our study.

Meanwhile, mounting evidence in line with our findings suggests that the prevalence of epithelial hyperplasia may increase with age and with BPH progression, and may be predominant in patients needing surgery for BPH. Histopathological examination of biopsies and TURP tissues from 210 patients with BPH revealed 91.4% cases of epithelial hyperplasia, and 8.6% cases of stromal hyperplasia [21]. Histopathological examination of tissues from 105 patients undergoing TURP revealed glandular-predominant histopathology in 58.1%, stromal-predominant histopathology in 20% and mixed histopathology in 21.9% of patients [22]. In tissues from 106 patients (97 from TURP), stromal hyperplasia predominated in only 3.8% of them [23]. In 567 patients undergoing TURP, epithelial hyperplasia predominated in 70−80% of all patients >50 years and still, though lower (60%) in younger patients (i.e., in an undefined number of patients <50 years undergoing TURP) [24]. A stromal/epithelial ratio of 4.65 was found in patients with rapid disease progression, needing surgery before 50 years of age, but of 2.03 in age-matched prostates and of 1.23 in elderly BPH patients (with 25 examined patients per group) [25]. Morphometrically assessed stromal/epithelial ratios in patients receiving surgery for BPH by TURP or open prostatectomy mounted to 2.6 in patients with hereditary BPH, and 2.6 in age-matched patients but 1.7 in prostate weight-matched patients, again undergoing surgery for BPH [26]. The ratios reported in our study are based on Ct values for calponin or cytokerain-19 assessed by RT-PCR, not allowing comparisons to morphometrically assessed stromal/epithelial ratios.

The conclusiveness of previous findings on tissue composition in BPH was repeatedly supposed to underlay restrictions [10]. Tissues from BPH without need of surgery or without severe symptoms are available only from surgery for prostate cancer or from biopsies in suspected prostate cancer. In turn, tissues without BPH are only accessible from autopsy, associated with all restrictions resulting from delays of tissue sampling [27]. Biopsies may be unsuitable for definite characterization of the predominant BPH phenotype, as they are too small to represent the whole prostate [28]. Apart from zonal differences in prostate pathology, BPH may occur in nodules, including nodules with either stromal or epithelial hyperplasia, both kinds of nodules (though one type may be predominant), and of course internodular tissue [10]. Similar limitations were still suspected to apply for TURP chips, which have been commonly used to study morphometry in BPH [10]. In contrast, samples in our study were pooled from each patient, i.e., about 5 tissue pieces from the same laser-enucleated patient were combined to one single sample for PCR, or about 10 pieces for Western blot analyses, which were then homogenized and analyzed together.

Cytokeratin-19 is expressed in all three types of epithelial cells in prostate glands, and in all conditions except cancer [11], despite variability of the glandular epithelium [10]. Thus, it is expressed in basal, luminal and atrophic epithelial cells, but not in stromal cells [11]. Other cytokeratins may be expressed as well in the glandular epithelium, but their expression may depend on cell type and on pathophysiological conditions [29,30]. Therefore, we decided to detect cytokeratin-19 as a marker, as this could consistently reflect the glandular content of tissues. The antibody used here is not specific for cytokeratin-19 but will probably react with any cytokeratin. Another cytokeratin in the non-malignant glandular epithelium is cytokeratin-18 [30,31]. Cytokeratin-19 and −18 have similar molecular weights (44 kDa, 48 kDa). Thus, both may have been detected at once in our Western blot analyses, explaining why bands were quite broad with this antibody. Notably, immunoreactivity with this antibody in human prostate tissues was strictly limited to glandular epithelia, and lacking in the stromal parts in fluorescence stainings in our previous studies [32–38]. Accordingly, this cytokeratin antibody appears suitable to assess the epithelial content in our samples. Calponin-1 in turn is expressed by any type of smooth muscle cells and required for contraction. It has been repeatedly used as a marker for smooth muscle cells in analyses of prostate tissues [39]. Immunoreactivity with the same antibody we used here was strictly limited to stromal compartments in fluorescence stainings of human prostate tissues in our previous studies [32–38]. Apart from cellular constituents, collagens and other extracellular matrix components may contribute as well to tissue composition and symptoms in prostates of patients needing BPH [40,41], which were not detected in our analyses.

While previous studies quantifying stromal and epithelial compartments addressed the prevalence of histological phenotypes in BPH or their relationships to prostate size, our study is one of few, if not the first to aim at relationships of tissue composition and voiding symptoms. Our finding that prostate size correlated inversely with calponin across all included patients, but differentially in catheterized and non-catheterized patients may reflect a possible heterogeneity within the studied population, i.e., among patients undergoing surgery for BPH. Obvious heterogeneity between patients with and without catheterization for urinary retention has been recently suggested as well [13]. Notably, however, IPSS and Q_max_ were not related to the prostate volume, at least in non-catheterized patients. Definite conclusions for the catheterized subgroup are not possible, as IPSS and Q_max_ were not available from most of these patients.

Our present study does not include long-term follow-up data to evaluate whether tissue phenotypes predict post-operative outcomes following laser enucleation. Thus, future prospective studies with systematic follow-up (≥12 months) are warranted to assess whether glandular-epithelial predominance can predict post-HoLEP/ThuLEP outcomes. Such investigations could guide personalized treatment decisions, including the selection of patients for early surgical intervention versus continued or alternative medical therapies. Additionally, correlating preoperative tissue phenotypes with improvements in IPSS and Q_max_ after surgery could further validate the clinical utility of phenotype-based stratification in BPH management.

## Conclusions

Voiding symptoms in patients undergoing laser enucleation for BPH aggravate with increasing keratin content. Symptoms in patients needing surgery for BPH depend rather on glandular-epithelial hyperplasia, but not on stromal hyperplasia, explaining why these patients may be refractory to treatment with α_1_-blockers.

## Supporting information

S1 Raw images Raw images of Western blots The file contains uncropped Western blots shown in Fig 2c, and all uncropped Western blots used for quantification shown in Fig 5, together with positions of molecular weight markers. (PDF)
