# Supplementary material for: Voiding symptoms aggravate with decreasing stromal/epithelial ratio and increasing glandular-epithelial content in patients undergoing laser enucleation for benign prostatic hyperplasia, independently from prostate size
Source: PLoS One. 2026 Mar 24;21(3):e0345306. doi: 10.1371/journal.pone.0345306 (PMC13012515; doi:10.1371/journal.pone.0345306)

Uncropped Western blots shown in Fig 2c
(all blots: next pages)

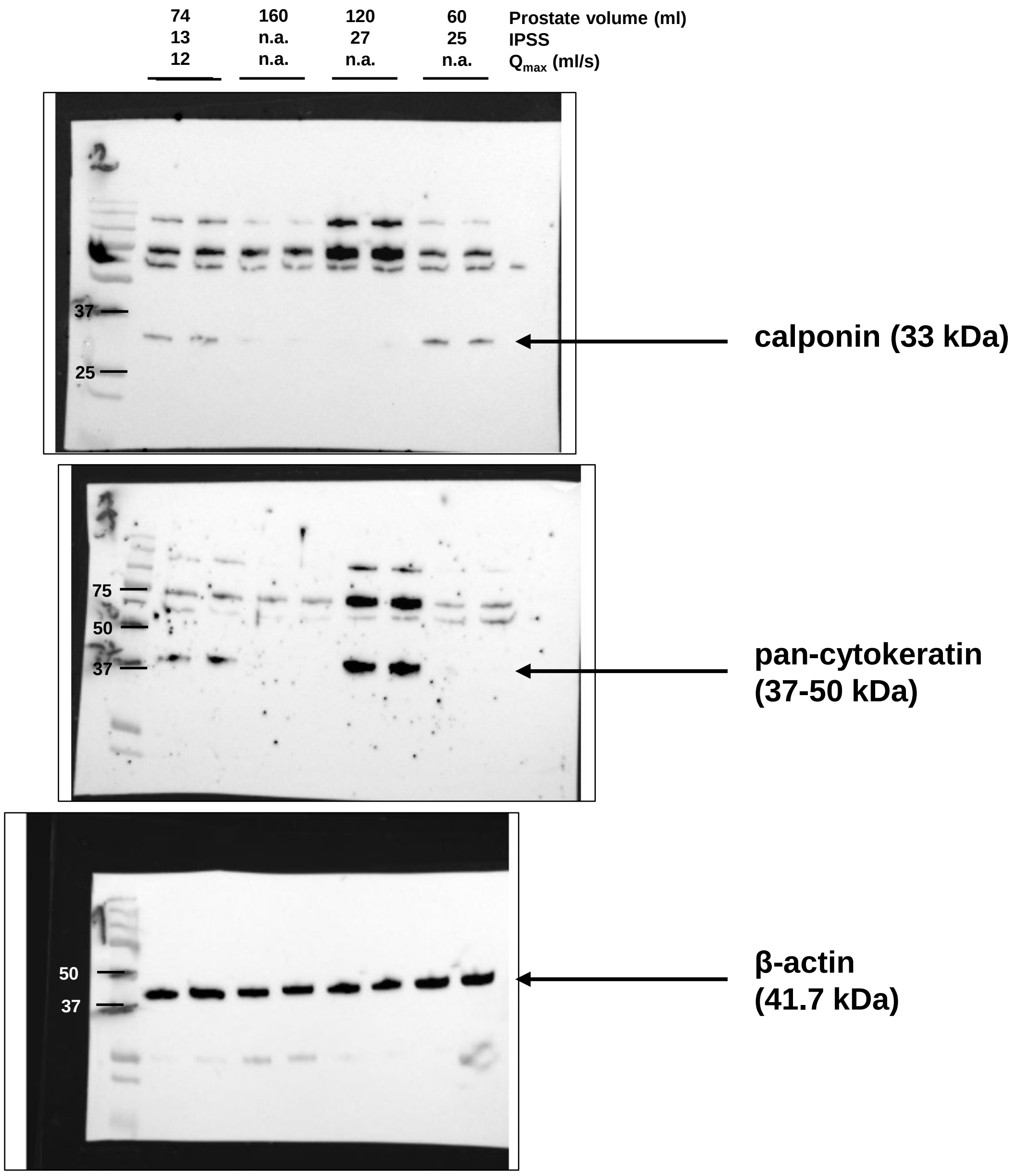

Fig 2c

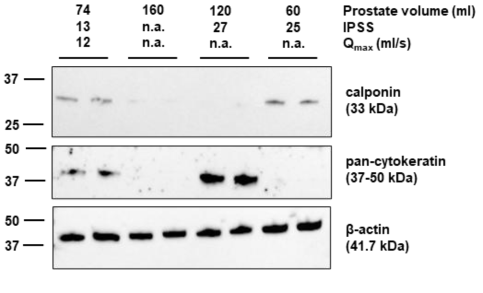

Notes:
Quantification was performed for bands matching
- 33 kDa for calponin
- 37-50 kDa for cytokeratin
- 42 kDa for β-actin

Uncropped blots used for quantification (Fig 5)
Patients‘ numbers indicated above the lanes are identical with those in correlation analysis (see files in public repository)

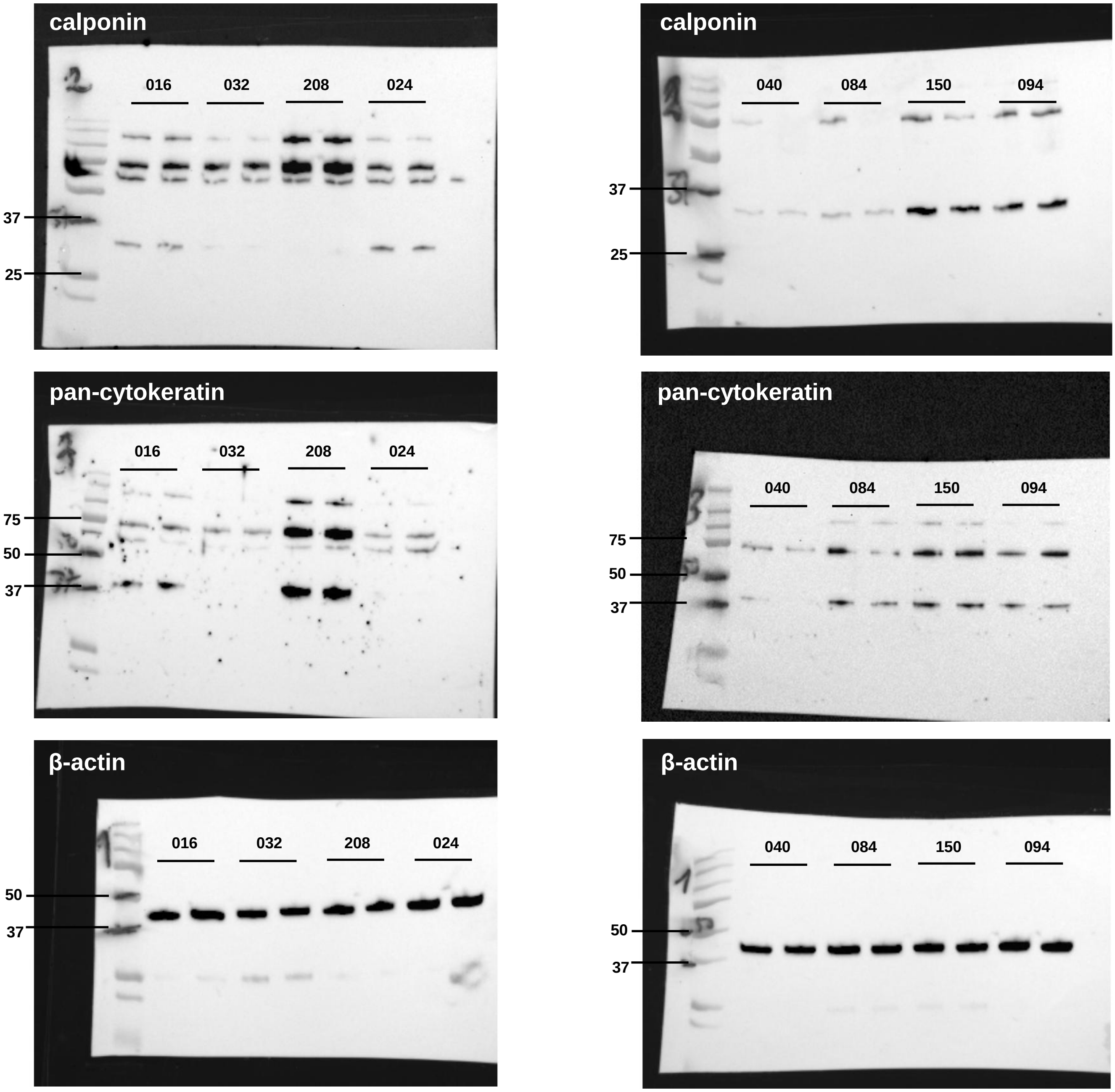

Uncropped blots used for quantification (Fig 5)
Patients‘ numbers indicated above the lanes are identical with those in correlation analysis (see files in public repository) (X, not included, unspecific)

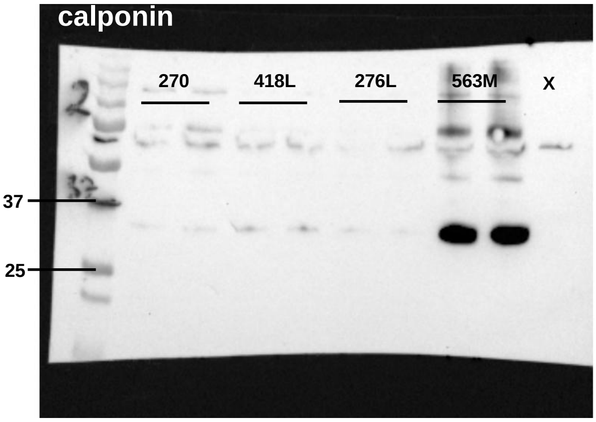

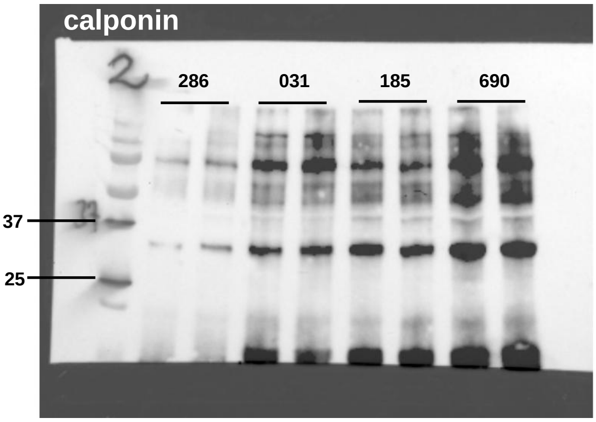

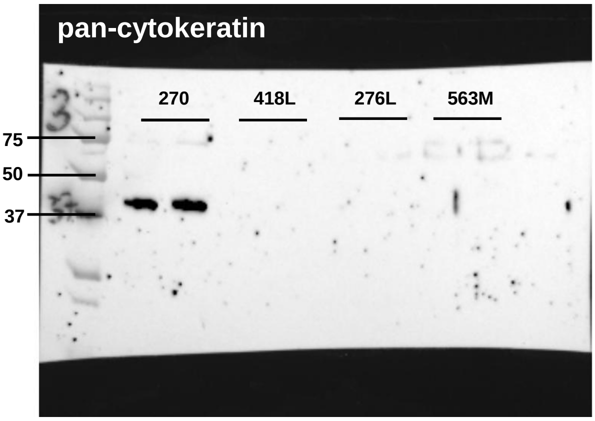

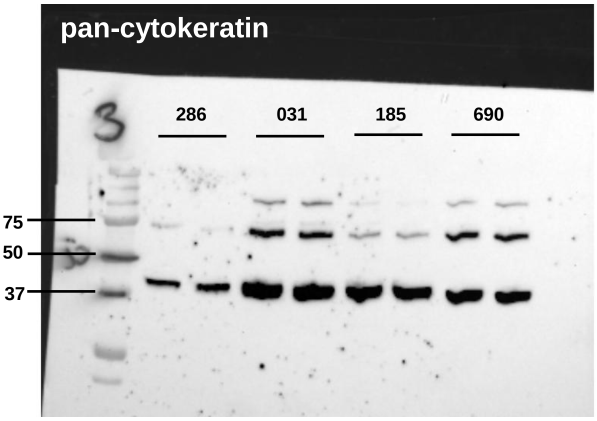

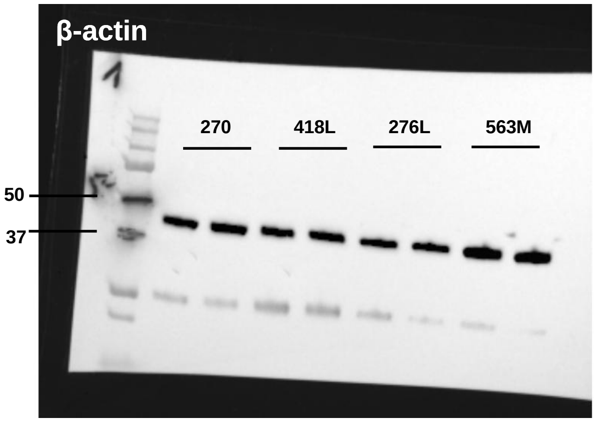

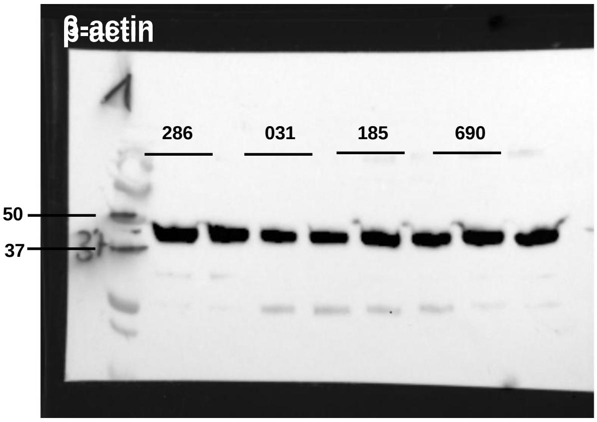

Uncropped blots used for quantification (Fig 5)
Patients‘ numbers indicated above the lanes are identical with those in correlation analysis (see files in public repository) (X, not included, unspecific)

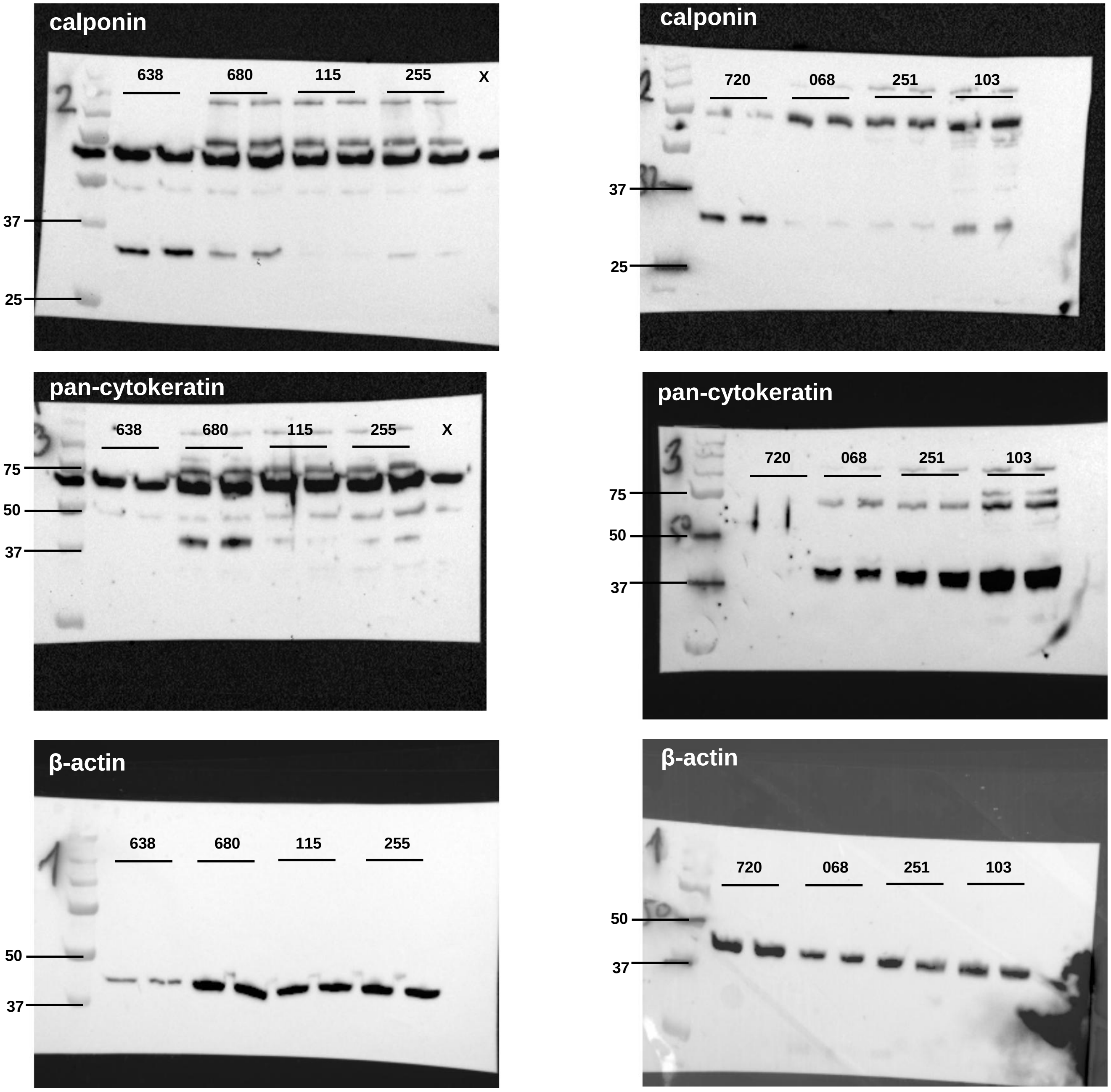

Uncropped blots used for quantification (Fig 5)
Patients‘ numbers indicated above the lanes are identical with those in correlation analysis (see files in public repository) (X, not included, unspecific)

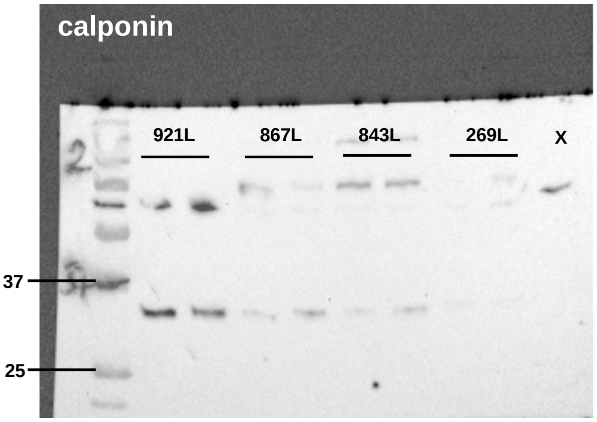

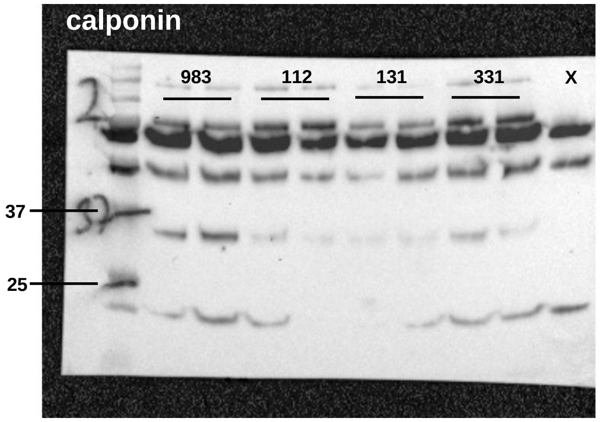

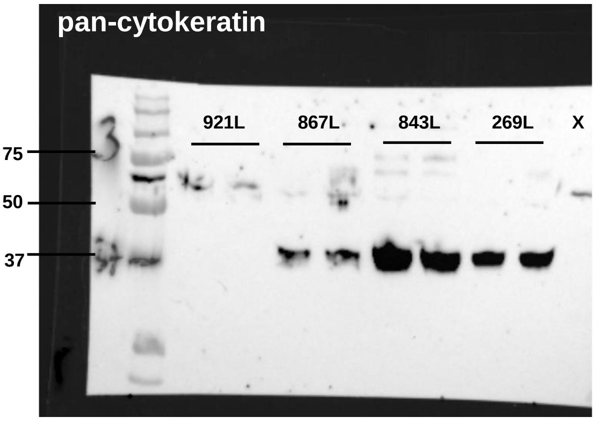

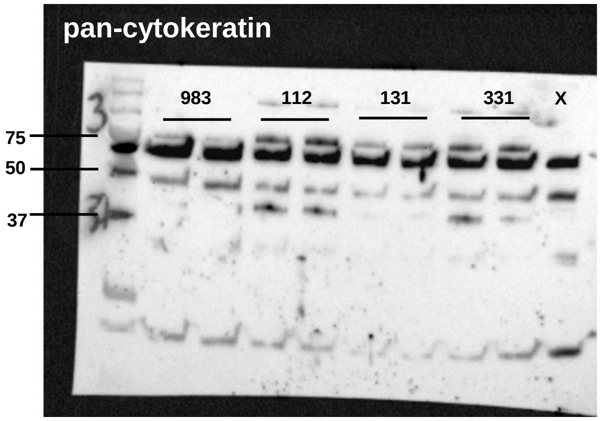

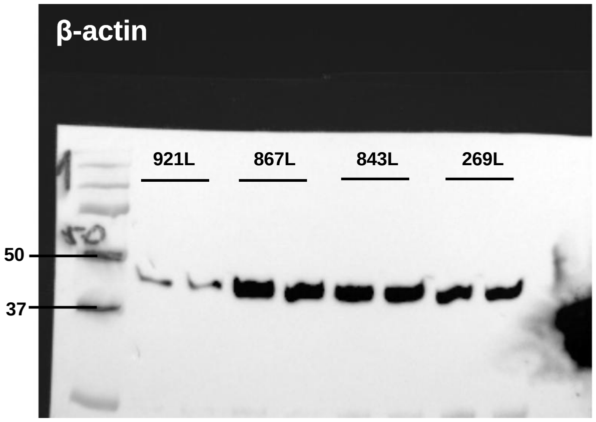

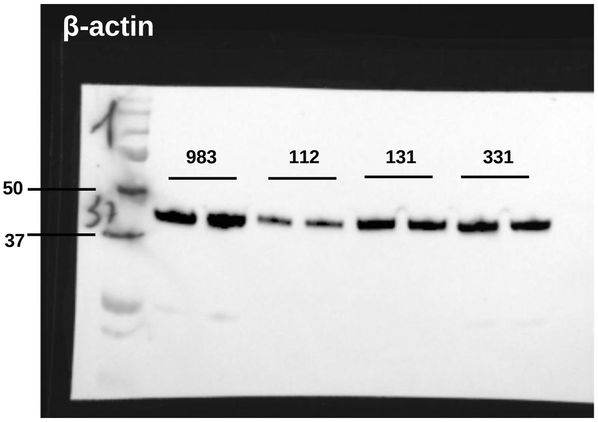

Uncropped blots used for quantification (Fig 5)
Patients‘ numbers indicated above the lanes are identical with those in correlation analysis (see files in public repository) (X, not included, unspecific)

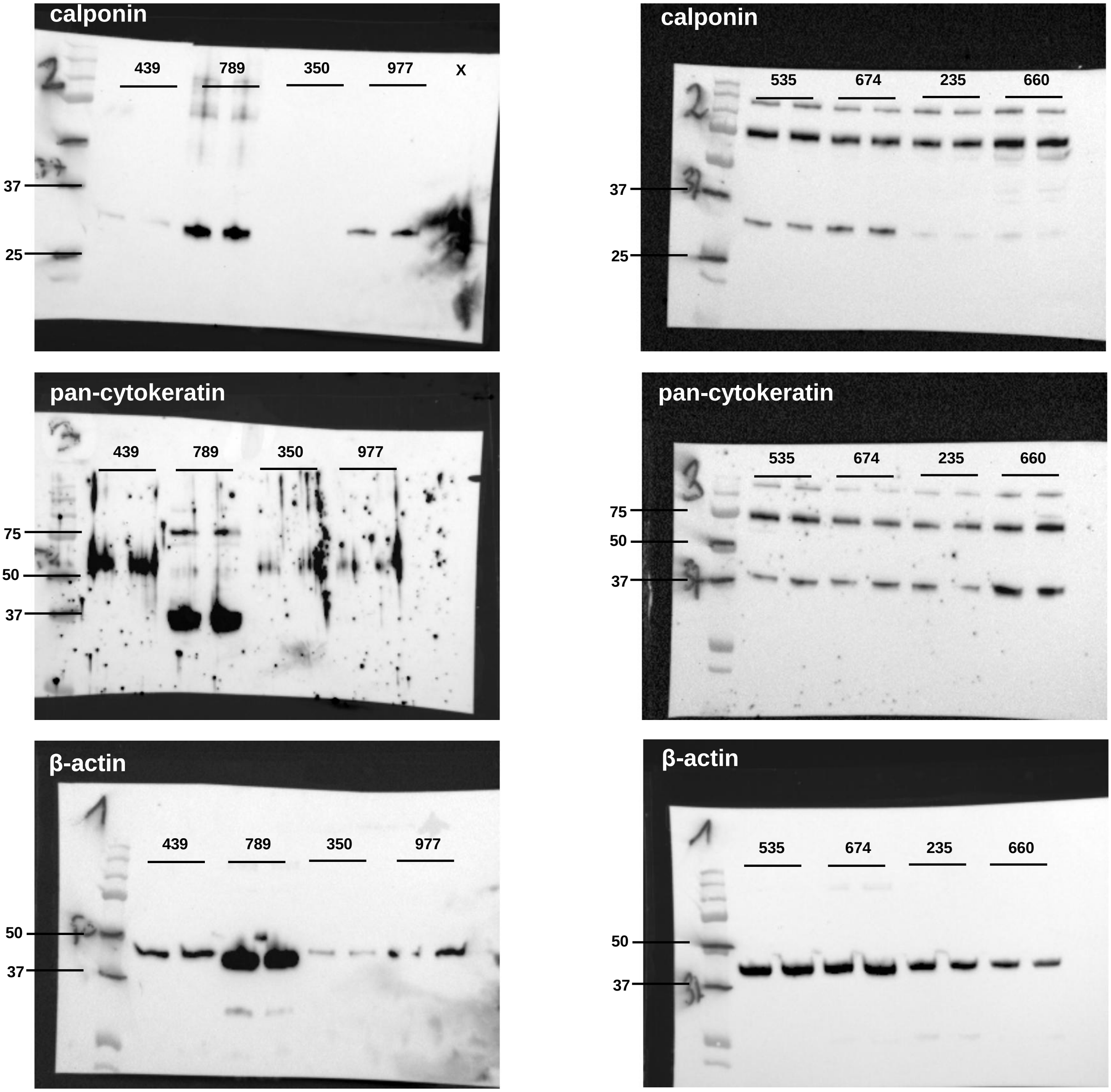

Uncropped blots used for quantification (Fig 5)
Patients‘ numbers indicated above the lanes are identical with those in correlation analysis (see files in public repository) (X, not included, unspecific; XX, not included, samples from TURP)

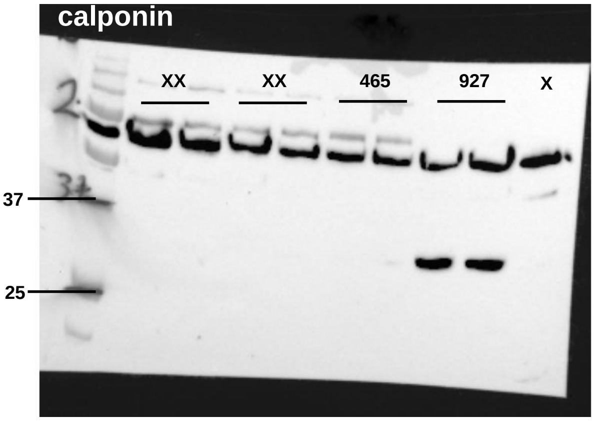

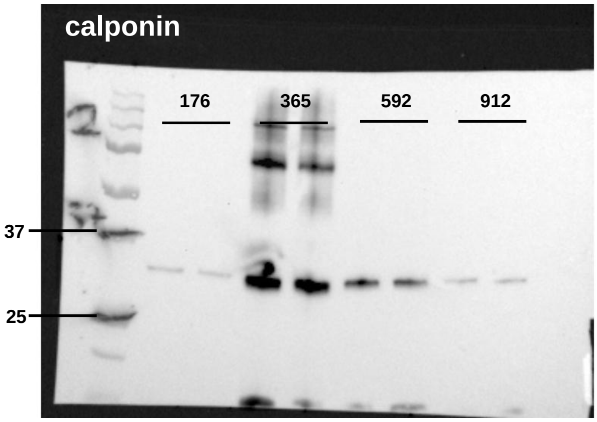

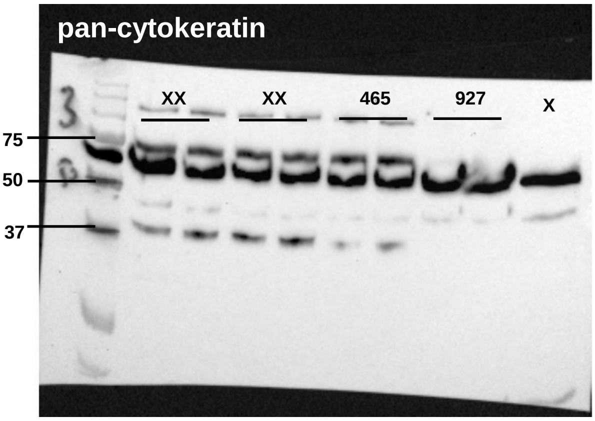

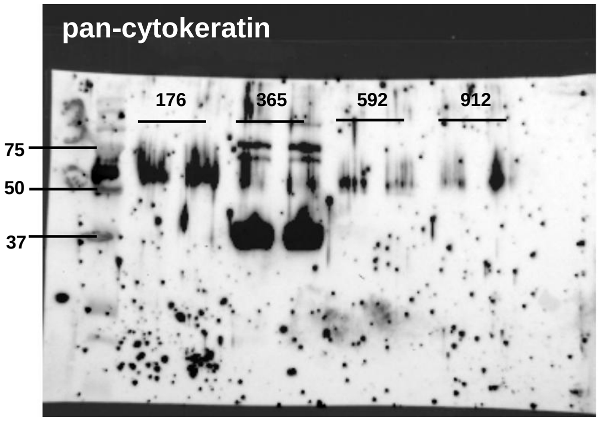

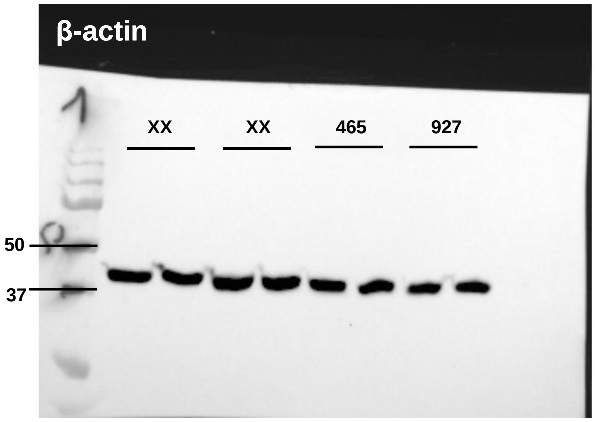

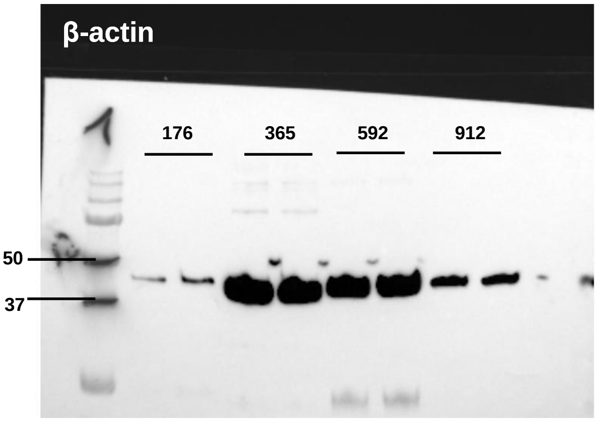

Supplement: S1 Raw images — (PDF) [file pone.0345306.s001.pdf]
